# Inverse kinematic total knee arthroplasty using conventional instrumentation restores constitutional coronal alignment

**DOI:** 10.1002/ksa.12306

**Published:** 2024-06-03

**Authors:** Sarah Keyes, Shane P. Russell, Zsolt Bertalan, James A. Harty

**Affiliations:** ^1^ South Infirmary Victoria University Hospital Cork Ireland; ^2^ Bon Secours Hospital Cork Cork Ireland; ^3^ Department of Orthopaedic Surgery University College Cork Cork Ireland; ^4^ Royal College of Surgeons in Ireland Dublin Ireland; ^5^ ImageBiopsy Lab Wien Austria

**Keywords:** conventionally instrumented TKA, coronal alignment, iKA, kinematic, navigated TKA, robotic TKA

## Abstract

**Purpose:**

Restricted inverse kinematic alignment (iKA) is a contemporary alignment strategy for total knee arthroplasty (TKA), commonly performed with robotic assistance. While superior clinical results are reported for kinematic‐type alignment strategies, registry data indicate no survivorship benefit for navigation or robotic assistance. This study aimed to determine the efficacy of an instrumented, restricted iKA technique for achieving patient‐specific alignment.

**Methods:**

Seventy‐nine patients undergoing 84 TKAs (five bilateral procedures) using an iKA technique were included for preoperative and postoperative lower limb alignment analysis. The mean age was 66.5 (range: 43–82) with 33 male and 51 female patients. Artificial intelligence was employed for radiographic measurements. Alignment profiles were classified using the Coronal Plane Alignment of the Knee (CPAK) system. Preoperative and postoperative alignment profiles were compared with subanalyses for preoperative valgus, neutral and varus profiles.

**Results:**

The mean joint‐line convergence angle (JLCA) reduced from 2.5° to −0.1° postoperatively. The mean lateral distal femoral angle (LDFA) remained unchanged postoperatively, while the mean medial proximal tibial angle (MPTA) increased by 2.5° (*p* = 0.001). By preservation of the LDFA and restoration of the MPTA, the mean hip knee ankle angle (HKA) moved through 3.5° varus to 1.2° valgus.

The CPAK system was used to visually depict changes in alignment profiles for preoperative valgus, neutral and varus knees; with 63% of patients observing an interval change in classification.

**Conclusion:**

Encouraged by the latest evidence supporting both conventional instrumentation and kinematic‐type TKA strategies, this study describes how a restricted, conventionally instrumented iKA technique may be utilised to restore constitutional lower limb alignment.

**Level of Evidence:**

Level III.

**Abbreviations**
CPAK: Coronal Plane Alignment of the Knee
HKA: hip knee ankle angle
iKA: inverse kinematic alignment
JLCA: joint‐line convergence angle
JLO: joint‐line obliquity
KA: kinematic alignment
LDFA: lateral distal femoral angle
MA: mechanical alignment
Max: mechanical axis
MPTA: medial proximal tibial angle
PROMS: patient reported outcome measures
TKA: total knee arthroplasty

## INTRODUCTION

To combat the much‐cited 10%–20% of patients that remain dissatisfied following total knee arthroplasty (TKA), surgeons have sought to improve outcomes by capitalising on advances in perioperative anaesthesia, rehabilitation protocols, implant design, implant fixation, surgical technique and surgical alignment strategies [6, 35]. Increasing evidence has also supported the shift from mechanical alignment (MA) towards individualised alignment strategies such as functional alignment, restricted kinematic alignment (rKA) and restricted inverse kinematic alignment (iKA) [35, 36, 37].

Both MA and KA may be achieved by conventional instrumentation or by technology assistance using navigation, robotics or image‐derived instrumentation (custom cutting blocks).

Since Freeman's 1973 description, the gold‐standard technique of MA has aimed for a ‘straight leg’ by performing femoral and tibial bony osteotomies perpendicular to the mechanical axis (MAx) of the lower limb [11, 23, 35]. The knee is then balanced, independent of knee phenotype, by sequential release of the soft tissue structures about the knee, resulting in potentially less favourable patient outcomes [23, 33, 34]. However, the hypothesised reduced shear forces and increased survivorship of mechanically aligned knees now seems to be dispelled [10, 16, 18, 19, 23, 26, 27, 32].

MA fails to account for the significant variability in constitutional lower limb alignment [3, 4, 7, 13, 15]. The utilisation of a general‐purpose, single‐target alignment strategy for all patients (despite historically imperfect outcomes and advances in: implant design, polyethylene manufacturing and understandings of knee motion) is being challenged [2, 20].

The classification of constitutional alignment to either varus, neutral or valgus is oversimplified, and variations of normal lower limb alignment phenotypes should be considered [12, 13, 14]. Hirschmann et al. observed 43 functional knee phenotypes [15]. For the most common knee phenotypes seen, MA strategies resulted in a significantly higher volume of bone resection [20, 28]. The Coronal Plane Alignment of Knee (CPAK) system describes a straightforward matrix for lower limb hip knee ankle angle (HKA) and joint‐line obliquity (JLO) variations [23]. Alignment profiles are classified into one of nine groups according to a valgus, neutral or varus HKA coincident with an apex superior, neutral or inferior JLO.

Kinematic‐type strategies aim to restore constitutional knee mechanics by restoring the three kinematic axes of normal knee motion and therefore the prearthritic lower limb alignment [16, 17, 18, 23]. Through a femur‐first philosophy, resections are performed so that the position of the prosthesis replicates prearthritic joint geometry, negating the need for soft tissue releases (a pure measured resection technique). The patient's constitutional mechanical HKA and JLO are thereby restored [9, 10].

iKA utilises a tibia‐first philosophy. Conversely to KA, the tibial resection determines the femoral resections and joint balance. The soft tissue envelope is preserved, and balance is achieved through cuts that restore native limb alignment and replicate native joint motion [35].

The latest findings from the American Joint Replacement Registry, ‘do not support an improvement over conventional TKA with robotic assistance’ for TKA survivorship [21]. In addition, the 2023 Australian Orthopaedic Association National Joint Replacement Registry now demonstrates no survivorship benefit for navigated or robotic TKAs and reduced survivorship for TKAs using Image‐Derived Instrumentation compared with conventionally instrumented procedures [31].

Conscious of: (1) The increasing evidence supporting kinematic‐type alignment strategies over MA and (2) the emerging metadata evidence demonstrating no benefit for navigation or robotic‐assisted TKA, the authors sought to explore how a conventionally instrumented iKA technique may be used to restore constitutional alignment.

The aim of this study was to determine the accuracy of a conventionally instrumented iKA technique for achieving patient‐specific alignment in TKA. It was hypothesised that constitutional, patient‐specific alignment may be achieved by iKA techniques without the need for navigation or robotic assistance.

## MATERIALS AND METHODS

Ethical approval was granted by the Clinical Research Ethics Committee of the Cork Teaching Hospitals, Ireland. Funding was received by the University College Cork Research Committee for radiographic analyses. All patients provided informed consent. This study adheres to the tenets of the Declaration of Helsinki.

### Study design

A retrospective cohort study design was employed to identify all patients who underwent primary TKA by iKA from 2021 to 2023 at an elective orthopaedic unit. A consecutive series of patients without exclusions was identified using local electronic records. While deciding to exclude children and complex primary cases such as patients with: preoperative ligamentous insufficiencies (e.g., medial collateral ligament rupture); hypermobility disorders; extreme contractures or diaphyseal deformities, none of the above were encountered.

Ninety‐nine consecutive patients were identified and included for preoperative and postoperative long‐leg radiographic analysis. Twenty patients were subsequently excluded due to inadequate available imaging (no pelvis to foot radiograph). Seventy‐nine patients were therefore included, with five having undergone bilateral TKA, allowing for 84 procedures to be included for final analysis. The mean age was 66.5 years (range 43–82). Thirty‐three patients were male and 51 female. Forty‐two were left TKAs and 42 right.

Patients were classified according to CPAK with 12 patients demonstrating preoperative valgus alignment (HKA < 2°), 22 neutral (HKA −2° to 2°) and 47 varus (HKA > 2°).

### Surgical technique

All patients included in this study underwent primary TKA by means of a conventionally instrumented, restricted, iKA technique using the *Attune Knee System* (DePuy Orthopedics). This was a single‐centre (BSHC), single surgeon (J. H.) study. The tibial osteotomy was restricted to a medial proximal tibial angle (MPTA) of 92°–84° in keeping with previously published iKA boundaries which have shown no adverse effects on implant survivorship and represent 93% of native Caucasian MPTAs [1, 14, 19, 29, 32, 35].

A variety of cemented, cementless, posterior stabilised, cruciate retaining, fixed and rotating platform prostheses were implanted all using the same principles of iKA, as described below. All patients had end‐stage arthritis preoperatively with typical radiographic changes such as narrowing or obliteration of the joint space, tibial‐sided bone loss and resultant varus or valgus HKA deformities.

After standard anaesthesia, supine positioning and application of a tourniquet for cemented prostheses, standard skin preparation and draping were completed. A customary midline incision and medial parapatellar arthrotomy were performed before the eversion of the patella.

**Figure 1:**
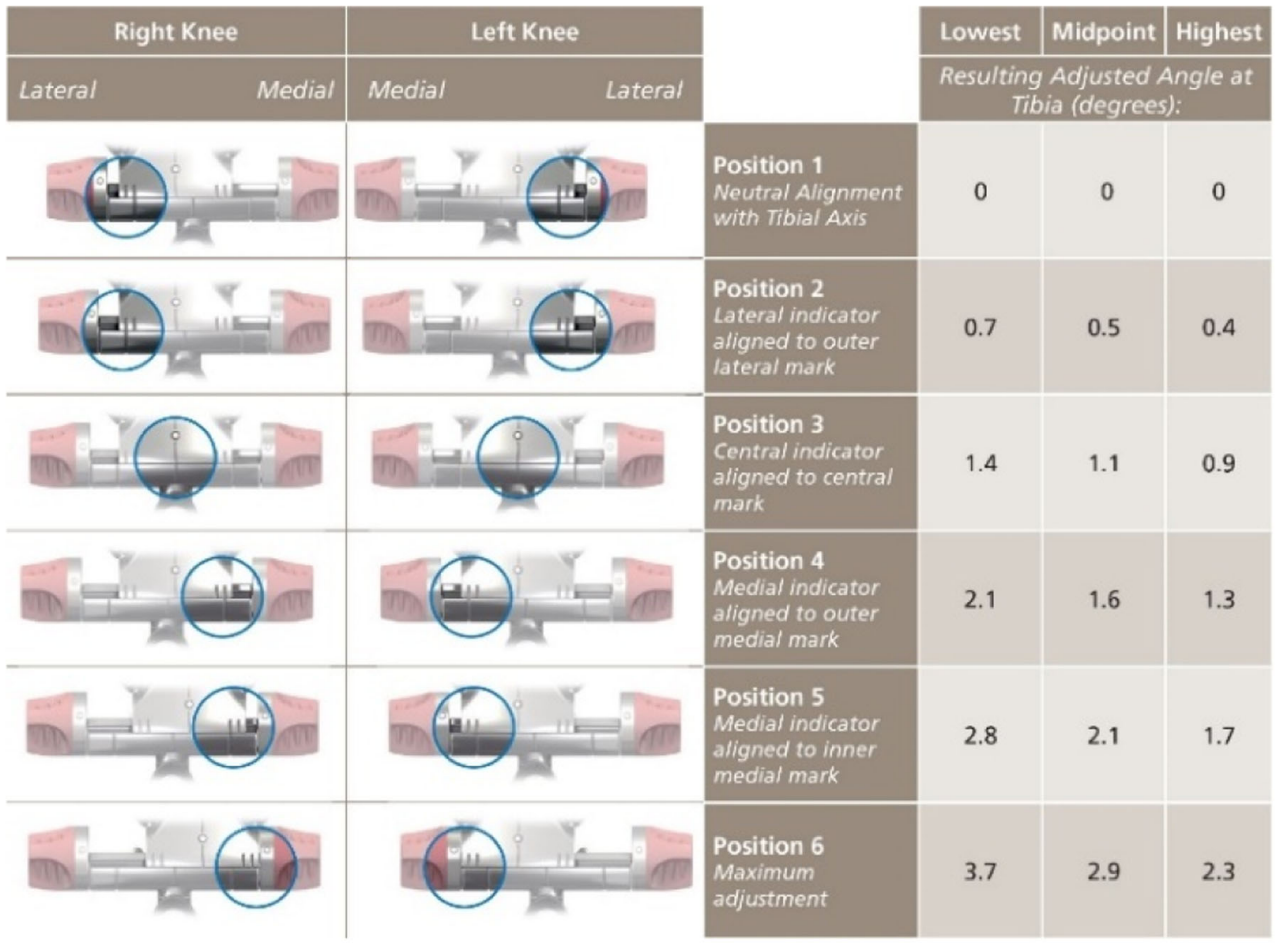
Tibial jig adjustment with corresponding planned MPTA (image license for use) [8].

A conservative distal femoral resection was first performed using an intramedullary jig, where the varus‐valgus position of the jig was set to resect 5 mm from an unworn medial or lateral femoral condyle or 3 mm from a bald condyle (accounting for 2 mm lost cartilage). A definitive tibial resection was then performed using an extramedullary guide, titrated as necessary to a varus‐valgus position that resected a measured 9 mm from an unworn medial or lateral plateau or 7 mm from a bald plateau. Where medial bone loss existed, appropriately less bone was resected to restore the native tibial joint line. A separate 2° varus tibial cutting block was available, if needed, to achieve an MPTA within the 5° restriction but beyond the jig capabilities (Figure 1).

A further 4 mm definitive distal femoral resection was then performed parallel to the tibial resection if the conservative extension gap was balanced. In rare cases where subtle balance adjustments were required, the coronal angle of the definitive distal femoral resection was fine‐tuned accordingly.

After femoral sizing, the flexion gap was symmetrically balanced, using the balancer‐sizer before the remaining femoral cuts were performed.

Femoral and tibial components were then trialed. The patella was resurfaced in all cases. Final femoral and tibial preparations were then performed before the implantation of components.

### Radiological measurement

**Figure 2:**
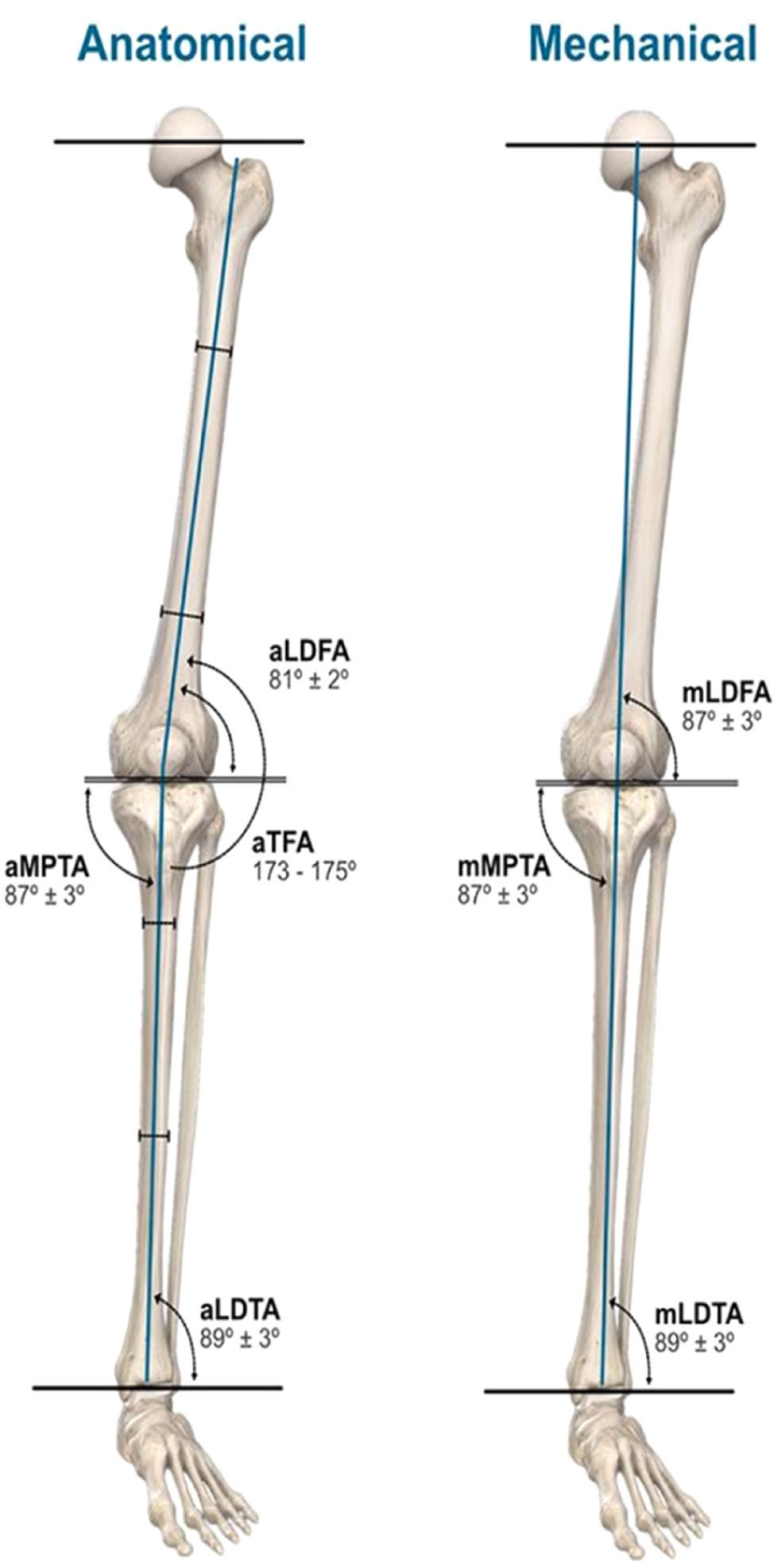
Angles measured by ImageBiopsy Lab.

All patients underwent preoperative and postoperative long‐leg radiographs using the ‘stand‐at‐attention’ position, with feet together and patellae orientated forward. After anonymisation, radiographic analysis was performed by a validated artificial intelligence method to calculate anatomic measurements of interest (96% reproducibility) (ImageBiopsy Lab GmbH) (Figure 2) [30].

The MAx of the femur was defined as a line from the centre of the femoral head to the centre of the distal femur. The MAx of the tibia was defined as a line at the midpoint of the tibia at the level of the knee joint to the centre of the tibial plafond at the ankle. The LDFA was defined as the lateral angle subtended by the MAx of the femur and the distal femoral joint line. The MPTA was defined as the angle subtended medially by the tibial MAx and the proximal tibial joint line. The HKA was calculated by the subtraction of the LDFA from the MPTA. The JLO was calculated by the addition of the LDFA and MPTA. The JLCA was calculated as the angle subtended by the distal femoral and distal tibial joint lines.

### Analysis

For the interpretation of results, the CPAK classification system was employed to demonstrate preoperative and postoperative lower limb alignments, with methods as described by MacDessi et al. [23].

Subanalyses were performed for patients presenting with the classically described ‘valgus’, ‘neutral’ or ‘varus’ arthritic patterns.

Statistical analysis was performed using Microsoft Excel with a paired *t* test *p* < 0.05 treated as significant.

## RESULTS

The mean HKA changed from 3.5° varus alignment to a mean of 1.2° valgus alignment (*p* < 0.001) (Table 1). To account for this, while the mean LDFA remained unchanged (87.5° preoperatively to 87.8° postoperatively, *p* = 0.39), the MPTA increased from 86.5° preoperatively to 89° postoperatively (*p* = <0.001). The arithmetic JLO therefore increased to 176.8° from 174°. The overall JLCA reduced from a mean of 2.5° preoperatively to −0.1° postoperatively (*p* = <0.001). Varus knees had a mean preoperative JLCA of 4.1° (range: 0.1°–8.4°), while valgus knees had a mean preoperative JLCA of −6.3° (range: −10.2 to −2.5).

**Table 1:** Results demonstrating mean preoperative and postoperative measurements in degrees, including the difference when comparing preoperative and postoperative data.

Angle (°)	Preop (σ, range)	Postop (σ, range)	Difference (σ, range)	*p* Value
HKA	3.5 (−10.2 to 13.8)	−1.2 (−10.2 to 5.1)	4.7 (−7 to 14.3)	<0.001
LDFA	87.5 (81 to 96.5)	87.8 (83 to 92.5)	0.3 (5.7 to −9.8)	0.39
MPTA	86.5 (77.6 to 92.4)	89 (83.9 to 95.9)	2.5 (11.2 to −3.5)	<0.001
JLO	174 (161.9 to 184.2)	176.8 (169.7 to 184.9)	6.7 (−0.2 to 17.2)	<0.001
JLCA	2.5 (−6.9 to 8.4)	−0.1 (−4.1 to 1.5)	2.6 (−6.4 to 9.2)	<0.001

Abbreviations: HKA, hip knee ankle angle; JLCA, joint‐line convergence angle; JLO, joint‐line obliquity.

**Figure 3:**
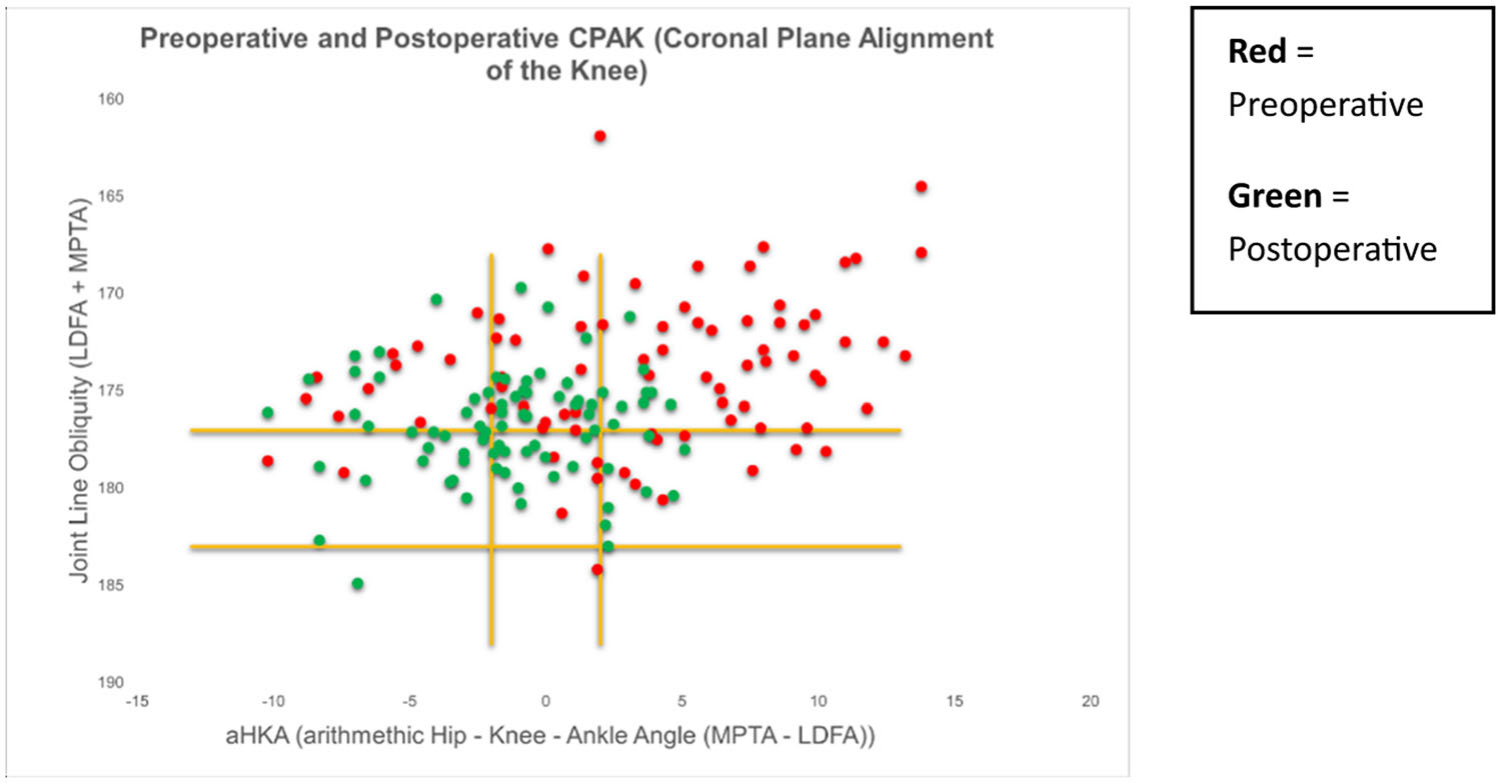
Scatter plot diagram demonstrating an overall trend towards neutral hip knee ankle angle (HKA) and horizontal joint‐line obliquity (JLO).

**Figure 4:**
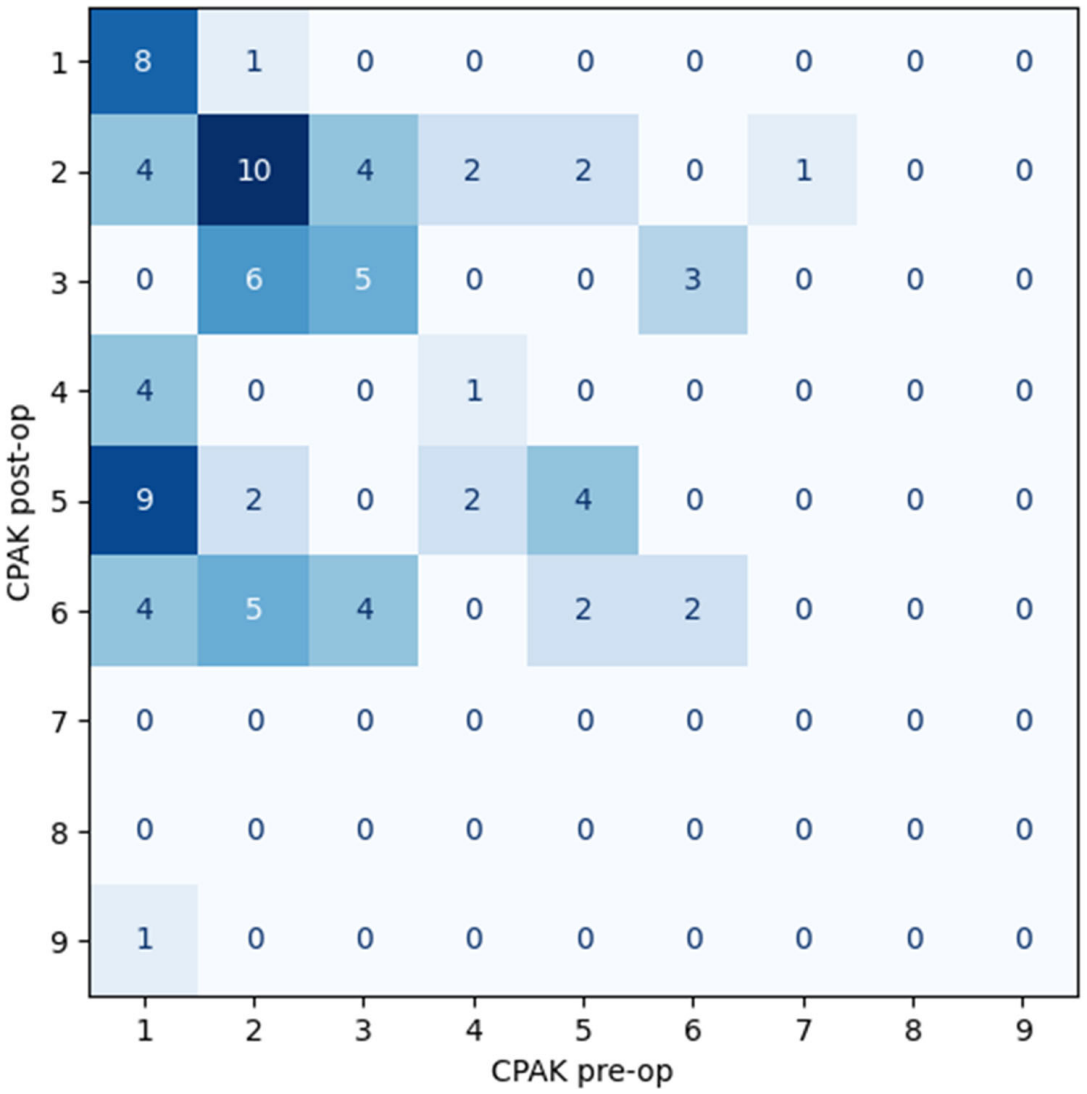
Confusion Matrix displaying Coronal Plane Alignment of the Knee (CPAK) preoperative classification on the X‐axis and CPAK postoperative category on the Y‐axis, where 37% of patients remained within the same CPAK classification.

There was an overall trend towards a neutral HKA and towards a horizontal JLO from preoperative to postoperative measurements (Figure 3). A total of 37% (*n* = 30) of patients remained within the same CPAK classification postoperatively (Figure 4).

**Figure 5:**
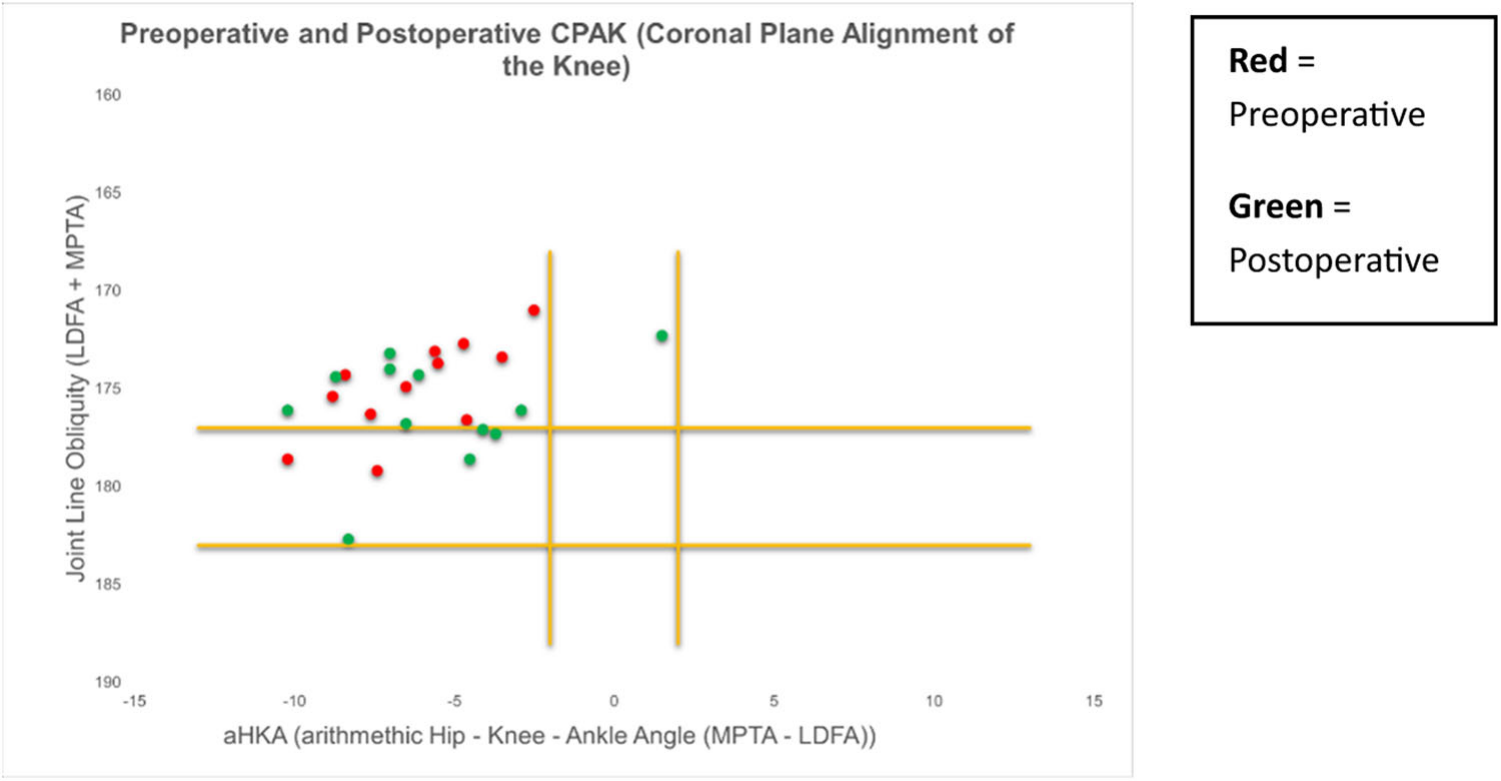
Scatter plot diagram demonstrating the 12 patients with a preoperative valgus alignment, where 11 remained in valgus, and one achieved neutral alignment.

**Figure 6:**
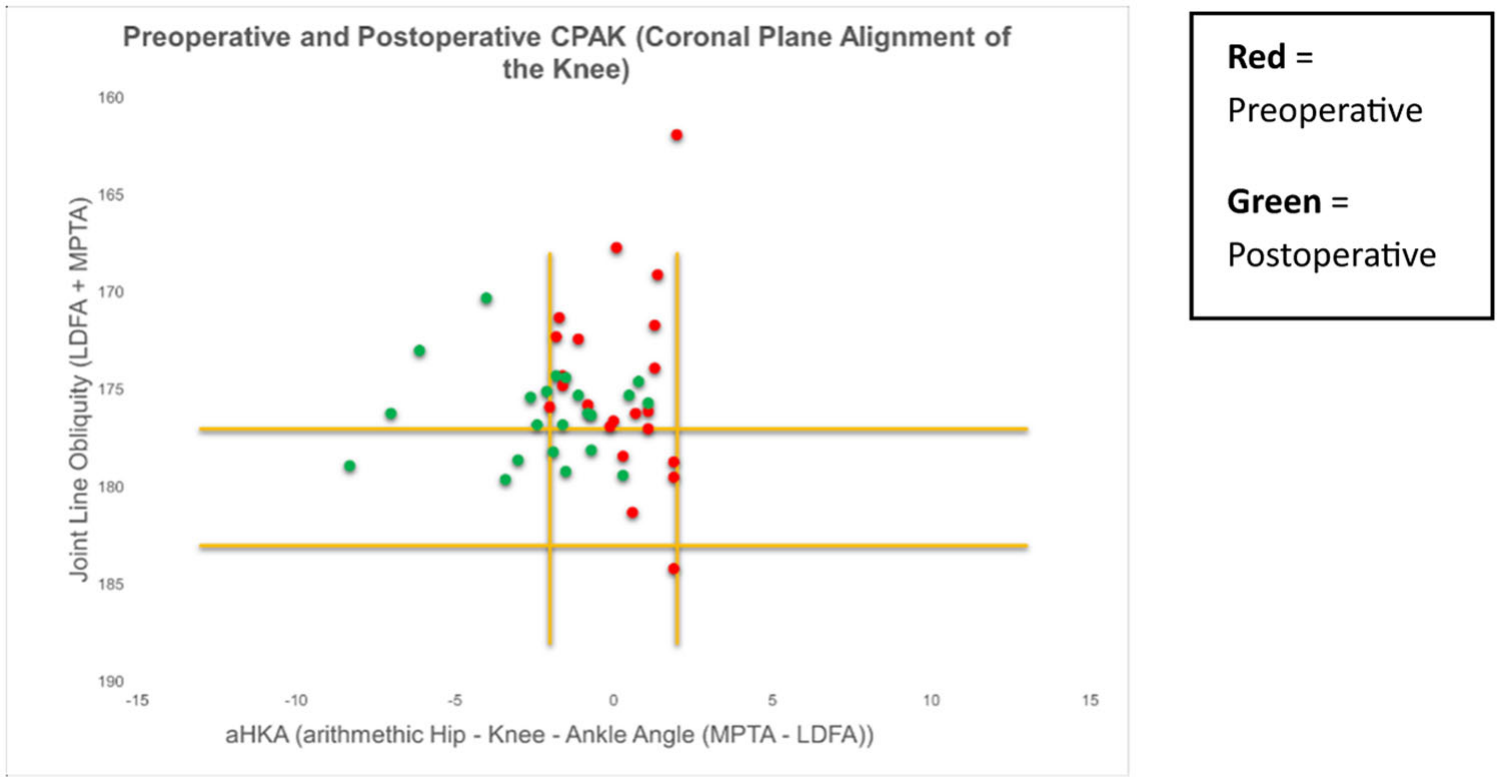
Scatter diagram demonstrating the 22 patients with a neutral hip knee ankle angle (HKA) preoperatively, where 14 remained in neutral and eight demonstrated postoperative valgus alignment.

**Figure 7:**
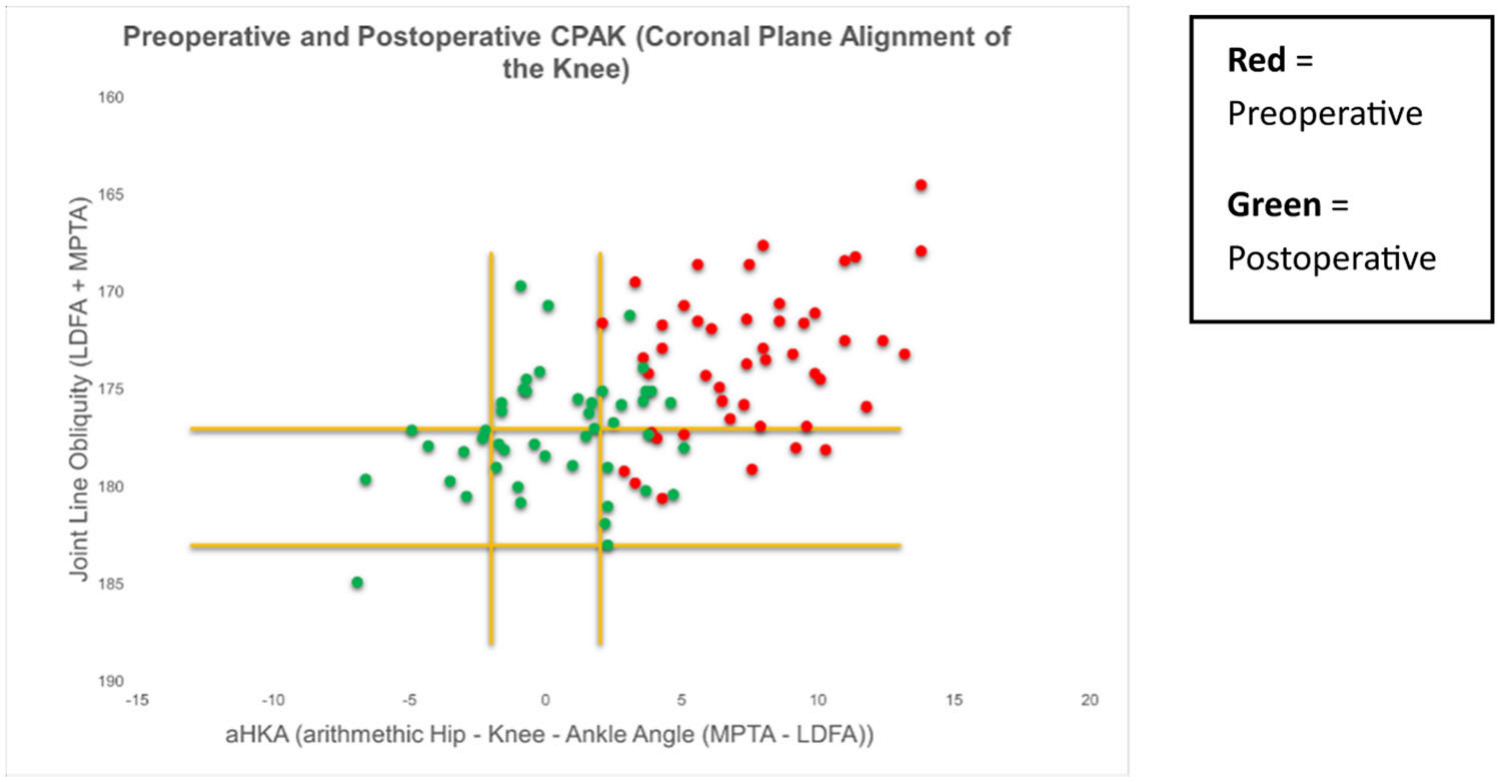
Scatter plot diagram demonstrating the 47 patients exhibiting varus alignment preoperatively, where nine displayed postoperative varus, 22 neutral and 16 remained in varus postoperatively.

Of the 12 patients with a preoperative valgus alignment, 11 remained valgus postoperatively while one patient achieved neutral alignment (Figure 5). Of the 22 patients with a preoperative neutral HKA, 14 remained neutral while eight demonstrated postoperative valgus alignment (Figure 6). The majority (*n* = 47) of patients exhibited preoperative varus alignment with nine of those demonstrating postoperative valgus, 22 neutral and 16 remaining genu varum postoperatively (Figure 7).

## DISCUSSION

This study demonstrates how the constitutional coronal plane alignment of the lower limb may be restored for patients undergoing TKA using a conventionally instrumented, restricted iKA technique.

As the overall cohort included patients with preoperative valgus, neutral and varus alignments, taking the mean values of the overall cohort result is not meaningful for the purposes of this study. Therefore, the interpretation of results is best observed when valgus, neutral and varus wear patterns are considered separately and the authors find the visualisation of the CPAK classification for each of these patient categories most useful (Figures 5, 6, 7).

The widened preoperative JLCA, ranging from −6.9° to 8.4°, best represents the preoperative asymmetry, or laxity, of the joint due to medial or lateral joint space narrowing, tibial bone loss and opposite side joint space opening. The principles of the described iKA technique are to restore joint surfaces to the prearthritic position, thereby restoring the HKA, LDFA, MPTA, JLCA and JLO to the native values. Native coronal values are exemplified where the JLCA approaches 0° when no soft tissue releases have been performed [3, 24]. Significant changes in CPAK classifications are achieved during restoration (Figure 2) [25].

As significant bone loss is less commonly observed at the femoral side but commonly seen at the tibial side, this study demonstrates how an iKA technique may be utilised to restore the HKA through preservation of the undeformed LDFA and restoration of the deformed MPTA. Using the described iKA technique, the distal femoral resection (effecting the postoperative LDFA) is performed after the proximal tibial resection (effecting the postoperative MPTA). It is therefore reassuring to observe an unchanged LDFA, as by proxy, this is an indication that the observed change in MPTA represents restoration of the joint line in the presence of preoperative bone loss.

Proponents of the KA alignment method may consider the ‘femur first’ technique to be advantageous as femoral bone loss is rarely significant. In addition, contrary to MA doctrine, kinematically aligned TKAs demonstrate lower intercompartmental pressure differences; reduce the need for intraoperative bony recuts; and reduce the incidence of tibiofemoral lift‐off (due to over‐tight contralateral compartments) [22, 23]. Kinematic‐type techniques have demonstrated a superior range of motion and superior clinical patient‐reported outcome measures (PROMS) compared with MA techniques in medium‐term follow‐up [9, 10, 34]. Unlike a kinematic tibial plateau resection, a kinematic distal femoral resection may be achieved without having to account for bone loss in addition to cartilaginous loss. However, this study now demonstrates how a native LDFA may be reliably achieved through a ‘tibia‐first’ technique by using iKA principles with accurate estimation of both cartilaginous and bony tibial plateau losses.

Orsi et al. compared robotic‐assisted iKA and gap‐balancing techniques, measuring CPAK outcomes [25]. The robotic iKA group observed more accurate restoration of the native MPTA for all patients and a more accurate LDFA restoration for varus and neutral knees. In comparison, the mean postoperative LDFA for this study also observed no significant change, while the MPTA underwent significant mean change during tibial restoration. Similarly to Orsi et al., this study observed the restoration of the JLO, crucial for kinematic optimisation [5]. In addition, similarly to Osri et al's findings where just 32% of the cohort observed no interval CPAK change, 39% of patients in this study observed no CPAK change. It is reassuring to identify similar results in the present study during the restoration of constitutional coronal alignment without robotic assistance.

DeGrave et al. also evaluated the effects of a robotic iKA technique on postoperative HKA and JLO, finding those with a preoperative varus deformity may benefit most from an iKA technique [36]. The results of the present study would support such a conclusion, where restoration of the native MPTA was successfully achieved for preoperatively genu varum knees.

Encouraged by the increasing evidence supporting kinematic‐type alignment strategies, this study demonstrates how an iKA technique, by conventional instrumentation, was successfully employed to achieve patient‐specific alignment.

This study had several limitations. First, by single‐centre, single‐surgeon design, the reproducibility of results has not been examined. However, controlling inter‐surgeon variability allowed for increased accuracy of reported results, where a stepwise, standardised technique is performed.

Second, while not examined in this study, further studies will report comparative PROMS, functional scores and long‐term outcomes.

Third, as predisease radiographs did not exist, the determination of true constitutional alignment was challenging. While consideration was given to employing the contralateral lower limb as a comparative control for prearthritic alignment, this was not possible due to the high rate of arthritis in the contralateral limb, the high prevalence of contralateral limb implants in our cohort, and indeed the paucity of literature evidence to support the assumption that native lower limb alignment profiles are symmetrical. However, as no soft tissue releases were performed and all resections were inside the ligamentous boundaries of the knee, the resulting balanced soft tissue envelope of the knee suggests the restoration of prearthritic ligamentous tension and therefore prearthritic lower limb alignment. The observed 0° mean JLCA is reassuringly supportive of a TKA well‐balanced by native constraints.

Lastly, no case of complex primary or revision TKAs were included in this study. In particular, no cases of hypermobility syndromes, preoperative ligamentous incompetence, extreme contractures or diaphyseal deformities were identified. The results of this study are therefore confined to the uncomplicated primary TKA.

## CONCLUSION

Encouraged by the latest evidence supporting both conventional instrumentation and kinematic alignment techniques for TKA, this study describes how a restricted, conventionally instrumented iKA technique can be utilised to successfully restore constitutional lower limb alignment. Further studies are required for comparison of instrumented and technology‐assisted iKA techniques.

## AUTHOR CONTRIBUTIONS

**Sarah Keyes**: Investigation; resources; data curation; writing—original draft; visualisation; project administration. **Shane P. Russell**: Methodology; formal analysis; data curation; writing—review & editing; supervision. **Zsolt Bertalan**: Resources; software; data curation. **James A. Harty**: Supervision; writing—review & editing; conceptualisation; funding acquisition.

## CONFLICT OF INTEREST STATEMENT

The authors declare no conflict of interest.

## ETHICS STATEMENT

Approval was obtained by the Clinical Research Ethics Committee, University College Cork and adheres to the tenets of the Declaration of Helsinki. Informed consent was provided by all participating patients in this study.

## Data Availability

The data that support the findings of this study are available on request from the corresponding author, JH. The data are not publicly available due to their containing information that could compromise the privacy of research participants.

## References

[1] Abdel, M.P. , Ollivier, M. , Parratte, S. , Trousdale, R.T. , Berry, D.J. & Pagnano, M.W. (2018) Effect of postoperative mechanical axis alignment on survival and functional outcomes of modern total knee arthroplasties with cement: a concise follow‐up at 20 years. Journal of Bone and Joint Surgery, 100, 472–478. Available from: 10.2106/JBJS.16.01587 29557863

[2] Beckers, G. , Meneghini, R.M. , Hirschmann, M.T. , Kostretzis, L. , Kiss, M.O. & Vendittoli, P.A. (2023) Ten flaws of systematic mechanical alignment total knee arthroplasty. The Journal of Arthroplasty, 39, 591–599. Available from: 10.1016/j.arth.2023.11.023 38007204

[3] Bellemans, J. , Colyn, W. , Vandenneucker, H. & Victor, J. (2012) The Chitranjan Ranawat award: is neutral mechanical alignment normal for all patients? The concept of constitutional varus. Clinical Orthopaedics & Related Research, 470, 45–53. Available from: 10.1007/s11999-011-1936-5 21656315 PMC3237976

[4] Bellemans, J. , Colyn, W. , Vandenneucker, H. & Victor, J. (2012) The Chitranjan Ranawat Award: is neutral mechanical alignment normal for all patients?: the concept of constitutional varus. Clinical Orthopaedics & Related Research, 470, 45–53. Available from: 10.1007/s11999-011-1936-5 21656315 PMC3237976

[5] Blakeney, W. , Clément, J. , Desmeules, F. , Hagemeister, N. , Rivière, C. & Vendittoli, P.A. (2019) Kinematic alignment in total knee arthroplasty better reproduces normal gait than mechanical alignment. Knee Surgery, Sports Traumatology, Arthroscopy, 27, 1410–1417. Available from: 10.1007/s00167-018-5174-1 30276435

[6] Bourne, R.B. , Chesworth, B.M. , Davis, A.M. , Mahomed, N.N. & Charron, K.D.J. (2010) Patient satisfaction after total knee arthroplasty: who is satisfied and who is not? Clinical Orthopaedics & Related Research, 468, 57–63. Available from: 10.1007/s11999-009-1119-9 19844772 PMC2795819

[7] Cooke, D. , Scudamore, A. , Li, J. , Wyss, U. , Bryant, T. & Costigan, P. (1997) Axial lower‐limb alignment: comparison of knee geometry in normal volunteers and osteoarthritis patients. Osteoarthritis and Cartilage, 5, 39–47. Available from: 10.1016/S1063-4584(97)80030-1 9010877

[8] CreativeCommons . (2024) Available from: creativecommons.org/licenses/by-nc/4.0/

[9] Dossett, H.G. , Estrada, N.A. , Swartz, G.J. , LeFevre, G.W. & Kwasman, B.G. (2014) A randomised controlled trial of kinematically and mechanically aligned total knee replacements. The Bone & Joint Journal, 96‐B, 907–913. Available from: 10.1302/0301-620X.96B7.32812 24986944

[10] Dossett, H.G. , Swartz, G.J. , Estrada, N.A. , LeFevre, G.W. & Kwasman, B.G. (2012) Kinematically versus mechanically aligned total knee arthroplasty. Orthopedics, 35, e160–e169. Available from: 10.3928/01477447-20120123-04 22310400

[11] Freeman, M.A.R. , Swanson, S.A.V. & Todd, R.C. (1973) Total Replacement of the knee Using the Freeman‐Swanson knee prosthesis. Clinical Orthopaedics and Related Research, 153–170. Available from: 10.1097/00003086-197307000-00020 4743445

[12] Hess, S. , Moser, L.B. , Robertson, E.L. , Behrend, H. , Amsler, F. , Iordache, E. et al. (2022) Osteoarthritic and non‐osteoarthritic patients show comparable coronal knee joint line orientations in a cross‐sectional study based on 3D reconstructed CT images. Knee Surgery, Sports Traumatology, Arthroscopy, 30, 407–418. Available from: 10.1007/s00167-021-06740-3 PMC8866364 34564737

[13] Hirschmann, M.T. , Hess, S. , Behrend, H. , Amsler, F. , Leclercq, V. & Moser, L.B. (2019) Phenotyping of hip–knee–ankle angle in young non‐osteoarthritic knees provides better understanding of native alignment variability. Knee Surgery, Sports Traumatology, Arthroscopy, 27, 1378–1384. Available from: 10.1007/s00167-019-05507-1 30968238

[14] Hirschmann, M.T. , Moser, L.B. , Amsler, F. , Behrend, H. , Leclercq, V. & Hess, S. (2019) Phenotyping the knee in young non‐osteoarthritic knees shows a wide distribution of femoral and tibial coronal alignment. Knee Surgery, Sports Traumatology, Arthroscopy, 27, 1385–1393. Available from: 10.1007/s00167-019-05508-0 30980119

[15] Hirschmann, M.T. , Moser, L.B. , Amsler, F. , Behrend, H. , Leclerq, V. & Hess, S. (2019) Functional knee phenotypes: a novel classification for phenotyping the coronal lower limb alignment based on the native alignment in young non‐osteoarthritic patients. Knee Surgery, Sports Traumatology, Arthroscopy, 27, 1394–1402. Available from: 10.1007/s00167-019-05509-z 30976825

[16] Howell, S.M. , Howell, S.J. , Kuznik, K.T. , Cohen, J. & Hull, M.L. (2013) Does a kinematically aligned total knee arthroplasty restore function without failure regardless of alignment category? Clinical Orthopaedics & Related Research, 471, 1000–1007. Available from: 10.1007/s11999-012-2613-z 22996362 PMC3563808

[17] Howell, S.M. , Hull, M.L. & Mahfouz, M. (2012) Kinematic alignment in total knee arthroplasty. Insall and scott surgery of the knee. Philadelphia, PA: Elsevier. pp. 1255–1268. Available from: 10.1302/2058-5241.5.200010

[18] Howell, S.M. , Papadopoulos, S. , Kuznik, K.T. & Hull, M.L. (2013) Accurate alignment and high function after kinematically aligned TKA performed with generic instruments. Knee Surgery, Sports Traumatology, Arthroscopy, 21, 2271–2280. Available from: 10.1007/s00167-013-2621-x 23948721

[19] Howell, S.M. , Shelton, T.J. & Hull, M.L. (2018) Implant survival and function ten years after kinematically aligned total knee arthroplasty. The Journal of Arthroplasty, 33, 3678–3684. Available from: 10.1016/j.arth.2018.07.020 30122435

[20] Karasavvidis, T. , Pagan Moldenhauer, C.A. , Lustig, S. , Vigdorchik, J.M. & Hirschmann, M.T. (2023) Definitions and consequences of current alignment techniques and phenotypes in total knee arthroplasty (TKA) ‐ there is no winner yet. Journal of Experimental Orthopaedics, 10, 120. Available from: 10.1186/s40634-023-00697-7 37991599 PMC10665290

[21] Kirchner, G.J. , Stambough, J.B. , Jimenez, E. & Nikkel, L.E. (2024) Robotic‐assisted TKA is not associated with decreased odds of early revision: an analysis of the American Joint Replacement Registry. Clinical Orthopaedics & Related Research, 482, 303–310. Available from: 10.1097/CORR.0000000000002783 37962943 PMC10776156

[22] MacDessi, S.J. , Griffiths‐Jones, W. , Chen, D.B. , Griffiths‐Jones, S. , Wood, J.A. , Diwan, A.D. et al. (2020) Restoring the constitutional alignment with a restrictive kinematic protocol improves quantitative soft‐tissue balance in total knee arthroplasty: a randomized controlled trial. The Bone & Joint Journal, 102, 117–124. Available from: 10.1302/0301-620X.102B1.BJJ-2019-0674.R2 31888372 PMC6974544

[23] MacDessi, S.J. , Griffiths‐Jones, W. , Harris, I.A. , Bellemans, J. & Chen, D.B. (2021) Coronal Plane Alignment of the Knee (CPAK) classification: a new system for describing knee phenotypes. The Bone & Joint Journal, 103‐B, 329–337. Available from: 10.1302/0301-620X.103B2.BJJ-2020-1050.R1 PMC7954147 33517740

[24] Micicoi, G. , Khakha, R. , Kley, K. , Wilson, A. , Cerciello, S. & Ollivier, M. (2020) Managing intra‐articular deformity in high Tibial osteotomy: a narrative review. Journal of Experimental Orthopaedics, 7, 65. Available from: 10.1186/s40634-020-00283-1 32902758 PMC7481321

[25] Orsi, A.D. , Wakelin, E. , Plaskos, C. , McMahon, S. & Coffey, S. (2023) Restricted inverse kinematic alignment better restores the native joint line orientation while achieving similar balance, laxity, and arithmetic hip‐knee‐ankle angle to gap balancing total knee arthroplasty. Arthroplasty Today, 19, 101090. Available from: 10.1016/j.artd.2022.101090 36688096 PMC9851873

[26] Parratte, S. , Pagnano, M.W. , Trousdale, R.T. & Berry, D.J. (2010) Effect of postoperative mechanical axis alignment on the fifteen‐year survival of modern, cemented total knee replacements. The Journal of Bone and Joint Surgery‐American Volume, 92, 2143–2149. Available from: 10.2106/JBJS.I.01398 20844155

[27] Rivière, C. , Iranpour, F. , Auvinet, E. , Howell, S. , Vendittoli, P.A. , Cobb, J. et al. (2017) Alignment options for total knee arthroplasty: a systematic review. Orthopaedics & Traumatology: Surgery & Research, 103, 1047–1056. Available from: 10.1016/j.otsr.2017.07.010 28864235

[28] Schelker, B.L. , Moret, C.S. , Sava, M.P. , von Eisenhart‐Rothe, R. , Graichen, H. , Arnold, M.P. et al. (2023) The impact of different alignment strategies on bone cuts in total knee arthroplasty for varus knee phenotypes. Knee Surgery, Sports Traumatology, Arthroscopy, 31, 1840–1850. Available from: 10.1007/s00167-023-07351-w PMC10089997 36811657

[29] Schelker, B.L. , Nowakowski, A.M. & Hirschmann, M.T. (2022) What is the “safe zone” for transition of coronal alignment from systematic to a more personalised one in total knee arthroplasty? A systematic review. Knee Surgery, Sports Traumatology, Arthroscopy, 30, 419–427. Available from: 10.1007/s00167-021-06811-5 PMC8866271 34973095

[30] Schwarz, G.M. , Simon, S. , Mitterer, J.A. , Frank, B.J.H. , Aichmair, A. , Dominkus, M. et al. (2022) Artificial intelligence enables reliable and standardized measurements of implant alignment in long leg radiographs with total knee arthroplasties. Knee Surgery, Sports Traumatology, Arthroscopy, 30, 2538–2547. Available from: 10.1007/s00167-022-07037-9 35819465

[31] Smith, P.N.G.D. , McAuliffe, M.J. , McDougall, C. , Stoney, J.D. , Vertullo, C.J. , Wall, C.J. , et al. (2023) Australian Orthopaedic Association National Joint Replacement Registry (AOANJRR). Hip, Knee & Shoulder Arthroplasty Annual Report, 2023, 6. Available from: 10.25310/YWQZ9375

[32] Vanlommel, L. , Vanlommel, J. , Claes, S. & Bellemans, J. (2013) Slight undercorrection following total knee arthroplasty results in superior clinical outcomes in varus knees. Knee Surgery, Sports Traumatology, Arthroscopy, 21, 2325–2330. Available from: 10.1007/s00167-013-2481-4 23552665

[33] Vigdorchik, J.M. , Wakelin, E.A. , Koenig, J.A. , Ponder, C.E. , Plaskos, C. , DeClaire, J.H. et al. (2022) Impact of component alignment and soft tissue release on 2‐year outcomes in total knee arthroplasty. The Journal of Arthroplasty, 37,2035–2040. Available from: 10.1016/j.arth.2022.04.042 35533822

[34] Wang, G. , Chen, L. , Luo, F. , Luo, J. & Xu, J. (2024) Superiority of kinematic alignment over mechanical alignment in total knee arthroplasty during medium‐ to long‐term follow‐up: a meta‐analysis and trial sequential analysis. Knee Surgery, Sports Traumatology, Arthroscopy, 32, 1240–1252. Available from: 10.1002/ksa.12093 38488220

[35] Winnock de Grave, P. , Kellens, J. , Luyckx, T. , Tampere, T. , Lacaze, F. & Claeys, K. (2022) Inverse kinematic alignment for total knee arthroplasty. Orthopaedics & Traumatology: Surgery & Research, 108, 103305. Available from: 10.1016/j.otsr.2022.103305 35513224

[36] Winnock de Grave, P. , Luyckx, T. , Claeys, K. , Tampere, T. , Kellens, J. , Müller, J. et al. (2022) Higher satisfaction after total knee arthroplasty using restricted inverse kinematic alignment compared to adjusted mechanical alignment. Knee Surgery, Sports Traumatology, Arthroscopy, 30, 488–499. Available from: 10.1007/s00167-020-06165-4 PMC8866329 32737528

[37] Winnock de Grave, P. , Luyckx, T. , Van Criekinge, T. , Müller, J.H. , Ollivier, B. , Van Eecke, E. et al. (2023) Inverse kinematic alignment accommodates native coronal knee alignment better in comparison to adjusted mechanical alignment and restricted kinematic alignment. Knee Surgery, Sports Traumatology, Arthroscopy, 31, 3765–3774. Available from: 10.1007/s00167-023-07326-x 36781450

